# Effect of spin-lock frequency on quantitative myocardial T1ρ mapping

**DOI:** 10.1186/s13244-024-01762-0

**Published:** 2024-07-12

**Authors:** Caiyun Han, Huimin Xu, Hui Gao, Fang Liu, Jian Wu, Yan Liu, Yong Cheng, Wei Deng, Xiuzheng Yue, Zhigang Wu, Yongqiang Yu, Ren Zhao, Yuchi Han, Xiaohu Li

**Affiliations:** 1 Department of Radiology, the First Affiliated Hospital of Anhui Medical University; Research Center of Clinical Medical Imaging; Anhui Province Clinical Image Quality Control Center, Hefei, 230032 Anhui Province China; 2 Department of Cardiology, the First Affiliated Hospital of Anhui Medical University, No. 218 Jixi Road, 230022 Hefei, China; 3 Philips Healthcare, Beijing, China; 4 Cardiovascular Division, Wexner Medical Center, College of Medicine, the Ohio State University Medical Center, Columbus, Ohio USA

**Keywords:** Magnetic resonance imaging, Myocardial, T1ρ mapping, Spin-lock frequency

## Abstract

**Objectives:**

To use T1ρ mapping to assess myocardial fibrosis and to provide a reference for future clinical application, it is necessary to understand the factors influencing T1ρ values. This study explored the influence of different spin-locking frequencies on T1ρ values under a 3.0-T MR system.

**Methods:**

Fifty-seven healthy subjects were prospectively and consecutively included in this study, and T1ρ mapping was performed on them in 3 short-axis slices with three spin-lock frequencies at the amplitude of 300 Hz, 400 Hz, and 500 Hz, then nine T1ρ images were acquired per subject. Four T1ρ-weighted images were acquired using a spin-lock preparation pulse with varying durations (0 msec, 13.3 msec, 26.6 msec, 40 msec). T1ρ relaxation times were quantified for each slice and each myocardial segment. The results were analyzed using Student’s t-test and one-way analysis of variance (ANOVA) methods.

**Results:**

Mean T1ρ relaxation times were 43.5 ± 2.8 msec at 300 Hz, 44.9 ± 3.6 msec at 400 Hz, and 46.2 ± 3.1 msec at 500 Hz, showing a significant progressive increase from low to high spin-lock frequency (300 Hz vs. 400 Hz, *p* = 0.046; 300 Hz vs. 500 Hz, *p* < 0.001; 400 Hz vs. 500 Hz, *p* = 0.043). In addition, The T1ρ values of females were significantly higher than those of males (300 Hz, *p* = 0.049; 400 Hz, *p* = 0.01; 500 Hz, *p* = 0.002).

**Conclusion:**

In this prospective study, myocardial T1ρ values for the specific CMR setting are provided, and we found that gender and spin-lock frequency can affect the T1ρ values.

**Critical relevance statement:**

T1ρ mapping could supersede late gadolinium enhancement for detection of myocardial fibrosis. Establishing reference mean values that take key technical elements into account will facilitate interpretation of data in disease states.

**Key Points:**

- This study established myocardial T1ρ reference values for different spin-lock frequencies.
- T1ρ values increased with spin-lock frequency, but numerical differences were minimal.
- Females had higher T1ρ values than males at all frequencies.

**Graphical Abstract:**

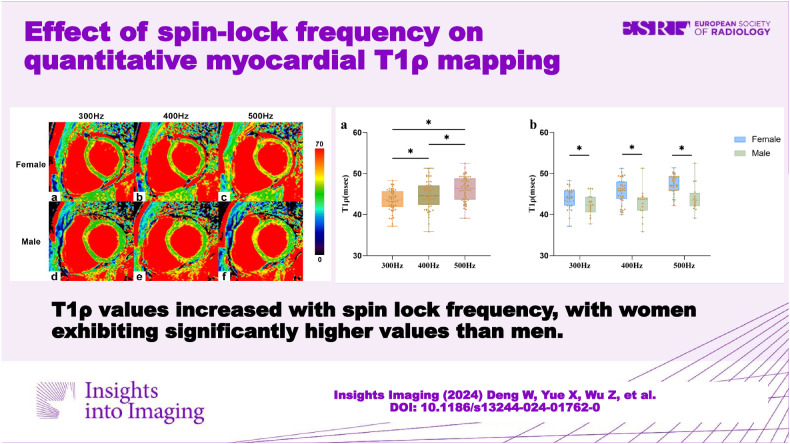

## Background

Myocardial fibrosis, a critical pathological process underlying ventricular remodeling, can be categorized into two subtypes: replacement and diffuse interstitial fibrosis [1]. The diffuse interstitial accumulation of fibrillar collagen is posited as a major determinant of left ventricular dysfunction and heart failure progression [2–4]. Therefore, sensitive and accurate methodologies for detecting and quantifying myocardial fibrosis are imperative for prognostication and gauging therapeutic efficacy in cardiac disease.

Cardiovascular magnetic resonance (CMR) imaging has emerged as a powerful tool for noninvasive quantification of myocardial fibrosis, with important implications for clinical decision-making. Late gadolinium enhancement (LGE) represents the gold standard for the assessment of replacement fibrosis [5, 6], providing unique insights into myocardial viability and distinguishing ischemic cardiomyopathy (ICM) from non-ischemic cardiomyopathy (NICM) [7–9]. However, LGE has limited sensitivity for diffuse interstitial fibrosis occurring at earlier disease stages. T1 mapping and extracellular volume fraction mapping allow quantification of diffuse fibrosis, complementing LGE [10], but demonstrate limited diagnostic accuracy [11]. T1ρ mapping represents a novel noninvasive cardiovascular magnetic resonance approach for quantitative evaluation of diffuse myocardial fibrosis, based on measurement of the transverse relaxation time constant in the rotating frame [12]. In contrast to conventional T1 and T2 mapping, T1ρ mapping employs spin-lock pulses selective for slower molecular motions, such as restricted protein dynamics, directly detecting myocardial fibrotic remodeling [12]. While originally studied in cartilage, intervertebral discs [13], and liver fibrosis [14], recent research demonstrates the utility of T1ρ mapping for assessment of both ICM [15] and NICM [16], highlighting the advantages of this quantitative technique for sensitive and specific quantification of myocardial fibrosis. Given the potential of T1ρ mapping to supersede late gadolinium enhancement for sensitive detection of myocardial fibrosis [17], optimization and standardization of the technique is imperative. Understanding factors influencing T1ρ values is critical, and previous studies have suggested that T1ρ values are inherently dependent on spin-lock frequency [17, 18]. It influences how effectively the imaging targets the interactions between water and macromolecules. The spin-lock frequency needs to be carefully chosen to optimize the contrast and sensitivity of the T1ρ signal to specific pathological changes, such as fibrosis, within the myocardium. However, the dependence of T1ρ relaxation times on spin-lock frequency has not been systematically studied, especially at 3.0 T field strengths. Therefore, this study aimed to investigate the effect of spin-lock frequency on myocardial T1ρ values and characterize the association between spin-lock frequency and T1ρ. Establishing reference mean values that takes key technical elements into account will facilitate the interpretation of data in disease states.

## Methods

### Study population

This prospective study enrolled 57 healthy adult volunteers at the First Affiliated Hospital of Anhui Medical University between July and September 2023. All participants provided written informed consent, and the institutional ethics committee approved the study protocol. Volunteers were consecutively recruited and comprised 18 males (mean age 26.4 ± 6.7 years) and 39 females (mean age 25.9 ± 6.6 years). To ensure volunteers were eligible for the study, all participants underwent a comprehensive health assessment prior to enrollment. This included: (1) Medical history questionnaire to screen for any pre-existing cardiovascular conditions, risk factors, or other relevant diseases. (2) Physical examination by a study physician, including heart auscultation, blood pressure measurement, and assessment for any concerning signs or symptoms. (3) A 12-lead electrocardiogram (ECG) was obtained and reviewed by a cardiologist to rule out any conduction abnormalities or other ECG findings that could indicate underlying heart disease. Only volunteers confirmed to be free of any overt cardiovascular disease or related risk factors, with normal screening results on history, exam and ECG, were found eligible and enrolled into the study.

### MR imaging protocol

CMR imaging was performed using a 3.0 T clinical scanner (Ingenia 3.0 T CX, Philips Healthcare, Best, the Netherlands) with a 32-element body coil. Subjects were scanned in the supine position with headfirst.

Cardiac gating was achieved using a wireless ECG device (Philips) with four MRI-compatible electrode pads on the left chest. Local B0 shimming optimized field homogeneity over the heart. T1ρ mapping was performed in 3 short-axis slices (basal, mid, and apical) using a T1ρ-prepared balanced steady-state free precession (bSSFP) sequence. Spin-lock pulses of 300 Hz, 400 Hz, and 500 Hz amplitude were applied within scanner-specific absorption rate limits, then nine T1ρ images were acquired per subject. The T1ρ spin-lock frequency order (300 Hz, 400 Hz, 500 Hz) was randomized for each participant. T1ρ maps were generated by fitting spin-lock times of 0, 13.3, 26.6, and 40 msec to a 3-parameter nonlinear least squares model using the Levenberg-Marquardt algorithm [19]. Image acquisition was performed in a single breath-hold per slice, with ECG triggering to minimize cardiac motion artifacts. The pulse sequence repetition time was three heartbeats, providing full slice coverage in 10 heartbeats (around 12 seconds). The T1ρ preparation scheme consisted of 90°x-SLy-180°x-SLy-90°x with refocusing pulses and dual spin-locks with opposite phases for B0/B1 correction [17]. Imaging parameters were: slice thickness 10 mm, matrix 180 × 148, bandwidth 2572 Hz/pixel, TR/TE 1.82/0.71 msec, FOV 360 × 297 mm^2^, flip angle 35°, SENSE factor 2, NSA 1, shot interval three heartbeats (Table 1).

**Table 1:** MR imaging parameters

Sequence	T1ρ mapping
FOV (mm²)	360 × 297
Slice thickness (mm)	10
Slice gap (mm)	12
Acquired pixel size (mm²)	2 × 2
Recon voxel size (mm²)	1.13 × 1.13
Matrix size	180 × 148
SENSE factor	2
TR (msec) / TE (msec)	1.82/0.71
Bandwidth (Hz / Pix)	2572
NSA	1
TSL (msec)	0, 13.3, 26.6, 40
Spin-locking frequency (Hz)	300, 400, 500
Shot interval	3 heartbeats
Scan time	10 heartbeats
Flip angle (°)	35

*FOV* field of view, *TR* time of repetition, *TE* time of echo, *NSA* number of signal averages, *TSL* time of spin locking

To assess scan-rescan reproducibility, ten volunteers underwent repeat CMR examination after exiting the scanner and resting briefly. Identical imaging protocols were utilized for the initial and repeat scans.

### Image processing and data analysis

Four different spin locking durations (TSL = 0, 13.3, 26.6, 40 msec) were used to calculate each T1ρ map using MATLAB (Version R2018b; MathWorks, Natick, MA). A monoexponential two-parameter decay model was applied using a Levenberg–Marquardt algorithm of nonlinear estimation to fit the T1ρ-relaxation time [19], which are described previously [20]. The left ventricular myocardium was divided into 16 segments per American Heart Association standards. Endocardial and epicardial borders were identified automatically and corrected manually. To minimize blood pool and epicardial fat effects, endocardial contours were shifted 10% outward and epicardial contours 10% inward. For images with motion artifacts, they were reviewed for quality, and rescanning was conducted when necessary to obtain images of adequate quality. T1ρ values were measured for each slice and segment. Short-axis cine images were analyzed using CVI42 software (Version 5.6.6, Circle Cardiovascular Imaging Inc., Calgary, Canada). They provided left ventricular ejection fraction (LVEF), cardiac index (CI), end-diastolic volume index (EDVi), end-systolic volume index (ESVi), stroke volume index (SVi), and mass index (Mi). Two observers (C.H. and H.X. both with 2 years’ experience in cardiac MRI) independently performed all analyses, blinded to each other’s results. A senior observer (X.L. with 15 years’ experience in cardiac MRI) adjudicated when there were discrepancies.

### Inter-and intraobserver reproducibility

Intra- and inter-observer reproducibility of T1ρ mapping was evaluated in a random subset of 20 participants. One observer (C.H.) performed T1ρ analysis for each left ventricular segment twice, with a minimum interval of 2 weeks between measurements. A second blinded observer (H.X.) independently analyzed the same cases to assess interobserver reproducibility.

### Statistical analyses

Statistical analysis was performed in SPSS 26.0 (IBM Corp, Armonk, NY), a post hoc power analysis was conducted using G*Power software (version 3.1.9.7). A *p* value < 0.05 was considered statistically significant. Data were assessed for normality using the Shapiro-Wilk test. Continuous normal variables were expressed as mean ± standard deviation (SD). Student’s t-test analyzed the differences in T1ρ values between males and females at each spin-lock frequency. One-way ANOVA evaluated differences in T1ρ values across spin-lock frequencies, with post-hoc pairwise comparisons using LSD tests with Bonferroni adjustment. Intra- and inter-observer reproducibility were assessed by intraclass correlation coefficients (ICCs) analysis. Scan-rescan reproducibility was evaluated by coefficient of variation (COV) and ICC.

## Results

### Baseline characteristics

Two subjects were excluded due to extensive image inhomogeneity or motion artifacts, leaving a final cohort of 57 subjects for analysis. Subjects were comprised of 18 males (20–49 years) and 39 females (mean age 20–60 years). Baseline characteristics are presented in Table 2. Due to the small sample size, no significant differences were observed across subjects in body mass index (BMI), heart rate (HR), LVEF, LVCI, or LVESVi. The LVEDVi, LVSVi, and LVMi were significantly higher in males than in females (all *p* < 0.05).

**Table 2:** Characteristics of the study population

	Total (*N* = 57)	Female (*N* = 39)	Male (*N* = 18)	*p* value
Age (years)	26.1 ± 6.6	25.9 ± 6.6	26.4 ± 6.7	0.814
Height (cm)	165.9 ± 8.2	161.6 ± 4.4	175.8 ± 6.0	< 0.001
Weight (kg)	59.2 ± 9.8	55.8 ± 7.3	66.9 ± 10.6	< 0.001
BSA (m²)	1.65 ± 0.17	1.58 ± 0.12	1.81 ± 0.17	< 0.001
BMI (kg/m²)	21.4 ± 2.5	21.3 ± 2.4	21.6 ± 2.6	0.760
HR (bmp)	73.4 ± 11.1	73.7 ± 11.4	72.9 ± 10.8	0.816
LVEF (%)	61.7 ± 6.0	61.6 ± 6.7	61.7 ± 4.4	0.962
LVCI (L/min/m²)	3.1 ± 0.6	3.1 ± 0.6	3.3 ± 0.5	0.234
LVEDVi (mL/m²)	72.9 ± 8.5	70.6 ± 8.1	78.0 ± 7.4	0.001
LVESVi (mL/m²)	28.2 ± 5.4	27.5 ± 5.9	29.8 ± 4.0	0.179
LVSVi (mL/m²)	45.0 ± 6.7	43.6 ± 6.6	48.2 ± 6.2	0.025
LVMi (g/m²)	38.2 ± 5.6	35.5 ± 4.1	44.3 ± 3.3	< 0.001

The comparison of study population characteristics between female and male. Data are presented as means ± SD. *p* value is for t-test between sexes. *BSA* body surface area, *BMI* body mass index, *HR* heart rate, *LV* left ventricle, *LVEF* LV ejection fraction, *LVCI* LV cardiac output index, *LVEDVi* LV end-diastolic volume index, *LVESVi* LV end-systolic volume index, *LVSVi* LV stroke volume index, *LVMi* LV mass index

### CMR T1rho measurements

**Fig. 1:**
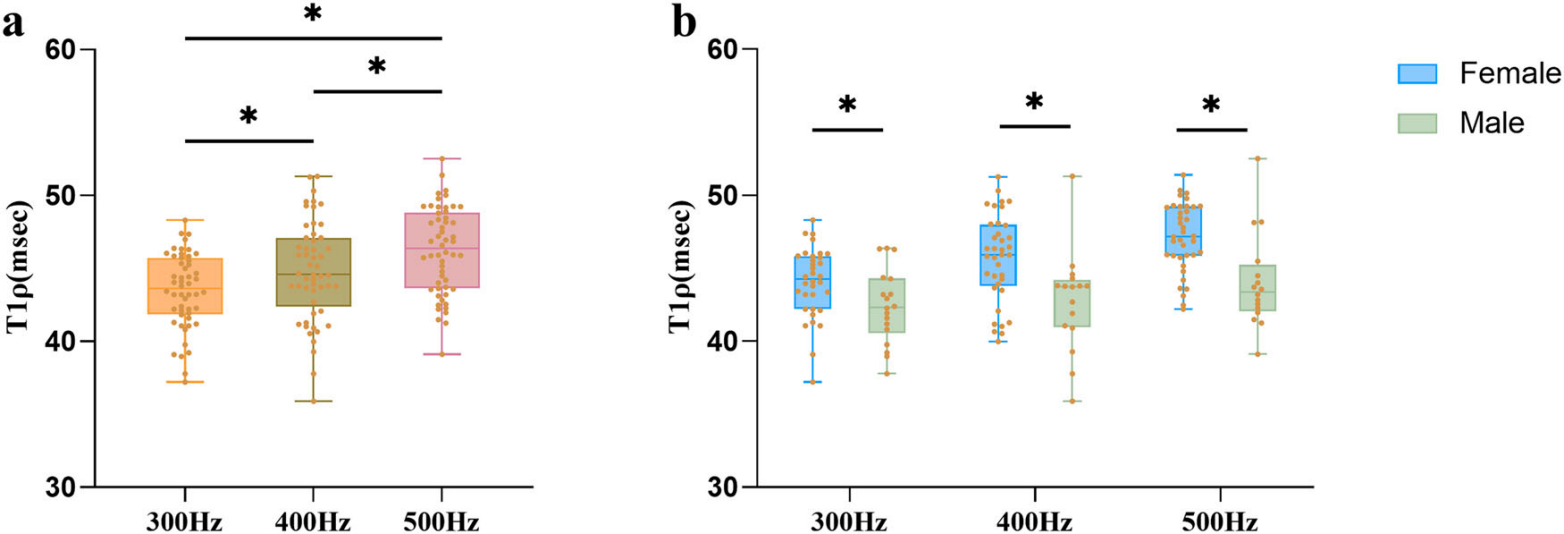
Comparison of T1ρ values in different spin-lock frequencies (**a**), comparison of T1ρ differences between females and males (**b**). Statistically significant differences are indicated by * on the box graphs. One-way ANOVA analysis was used for comparing the differences among the three spin-lock frequencies. Differences between females and males were tested using Student’s t-test

**Fig. 2:**
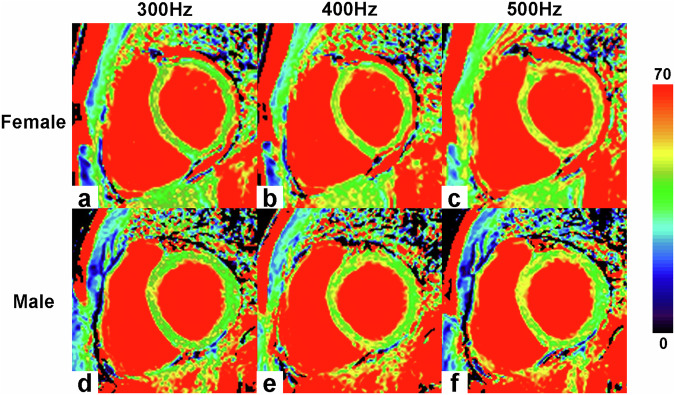
A 25-year-old female volunteer had T1ρ values of (43.6 ± 2.9 msec) (**a**), (45.0 ± 3.1 msec) (**b**), and (46.5 ± 3.4 msec) (**c**) at 300 Hz, 400 Hz, and 500 Hz, respectively; a 24-year-old male volunteer mean T1ρ values of (42.3 ± 3.0 msec) (**d**), (43.1 ± 3.4 msec) (**e**), and (44.9 ± 3.3 msec) (**f**) at 300 Hz, 400 Hz, and 500 Hz, respectively

**Fig. 3:**
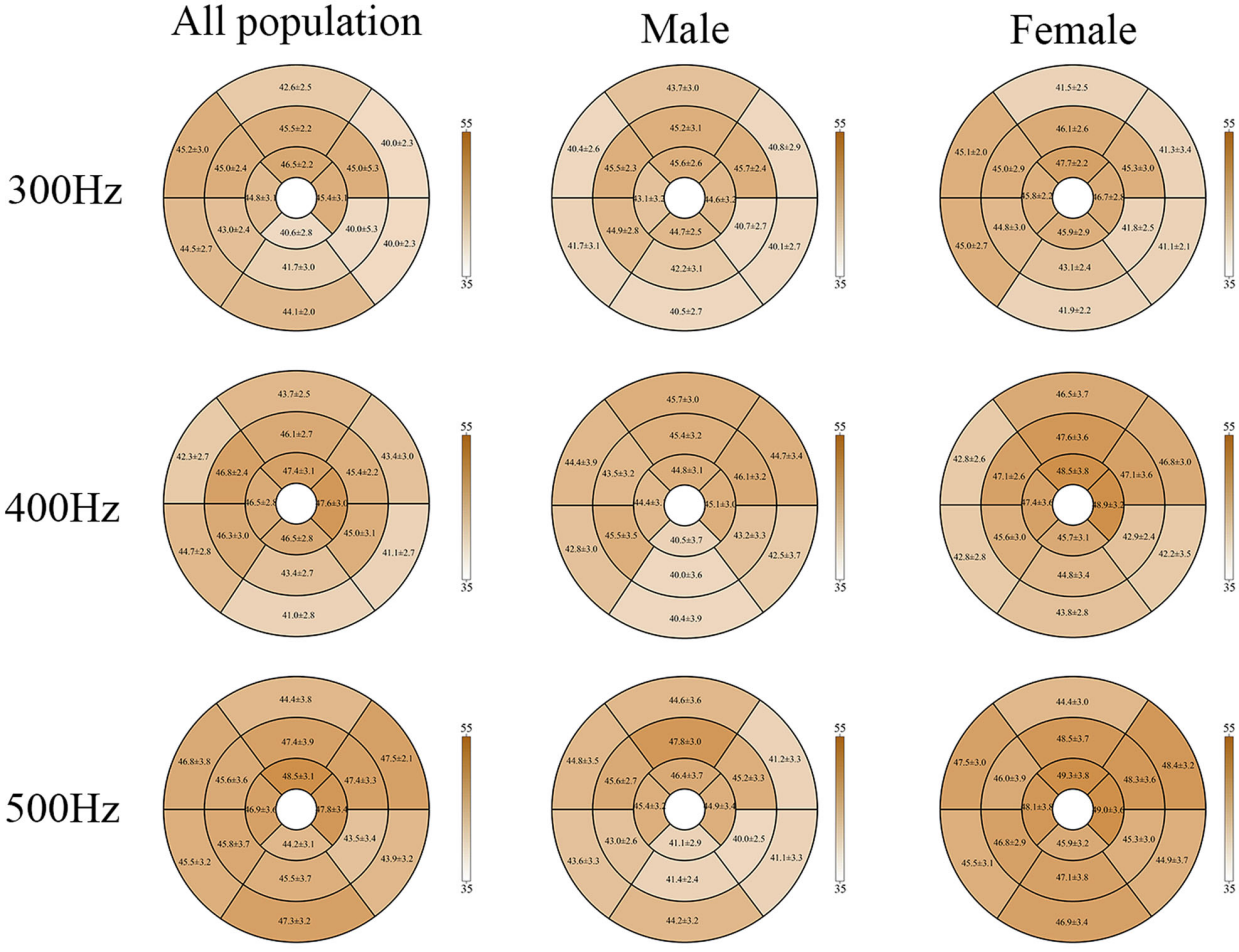
The T1ρ values of 16 segments of myocardium (without apex)

Of 2736 total myocardial segments, 2632 (96.2%) were included in the analysis after excluding 104 segments (3.8%), with artifacts predominantly located in the anterior, anteroseptal, and lateral walls. T1ρ relaxation times at the three spin-lock frequencies are presented in Table 3. T1ρ values increased progressively from lower to higher spin-lock frequencies (300 Hz vs. 400 Hz, *p* = 0.046; 300 Hz vs 500 Hz, *p* < 0.001; 400 Hz vs. 500 Hz, *p* = 0.043) (Fig. 1). Example T1ρ maps of male and female subjects are shown in Fig. 2. At 300 Hz, the T1ρ value was 43.5 ± 2.8 msec, with significantly higher values in females (44.1 ± 2.6 msec) compared to males (42.4 ± 2.8 msec) (*p* = 0.049). At 400 Hz, the T1ρ value was 44.9 ± 3.6 msec, again significantly higher in females (45.7 ± 3.2 msec) versus males (42.7 ± 3.9 msec) (*p* = 0.01). At 500 Hz, the T1ρ value was 46.2 ± 3.1 msec, with females (47.1 ± 2.5 msec) significantly exceeding males (44.0 ± 3.6 msec) (*p* = 0.002) (Fig. 1). The T1ρ values of the sex-specific myocardial 16-AHA segments are shown in Fig. 3.

**Table 3:** Results of T1ρ values of study population

	Total (*N* = 57)	Female (*N* = 39)	Male (*N* = 18)	*p* value
T1ρ (300 Hz) (msec)	43.5 ± 2.8	44.1 ± 2.6	42.4 ± 2.8	0.049
T1ρ (400 Hz) (msec)	44.9 ± 3.6	45.7 ± 3.2	42.7 ± 3.9	0.01
T1ρ (500 Hz) (msec)	46.2 ± 3.1	47.1 ± 2.5	44.0 ± 3.6	0.002

The comparison of T1ρ (300 Hz), T1ρ (400 Hz), T1ρ (500 Hz) values between females and males. Data are presented as means ± SD. *p* value is for t-test between sexes

### Inter-and intraobserver reproducibility

The measurement of T1ρ values showed a high intra- and interobservers reproducibility. The ICC values in Inter-observer reproducibility for T1ρ values at spin-lock pulse amplitudes of 300 Hz, 400 Hz, and 500 Hz were 0.881 (95%CI: 0.767–0.934), 0.917 (95%CI: 0.904–0.945), and 0.933 (95%CI: 0.879–0.969), respectively. The ICC values in Intra-observer reproducibility for T1ρ values at spin-lock pulse amplitudes of 300 Hz, 400 Hz, and 500 Hz were 0.889 (95%CI: 0.776–0.941), 0.928 (95%CI: 0.911–0.950), and 0.946 (95%CI: 0.885–0.960), respectively.

### Scan–scan reproducibility

Ten subjects were scanned twice, with 960 total segments reviewed and 924 evaluable segments analyzed in the different reproducibility assessments. The T1ρ values at spin-lock pulse amplitudes of 300 Hz, 400 Hz, and 500 Hz had the COV of 2.4%, 2.1%, and 1.8%, respectively, and the ICC of 0.891 (95%CI: 0.854–0.938), 0.905 (95%CI: 0.861–0.939), 0.911 (95%CI: 0.899–0.953), respectively.

### Post hoc power analysis

A post-hoc power analysis shows that the study had adequate statistical power given the sample size of 57 participants. The effect size in this study was 0.34, calculated from the mean and standard deviation of the primary outcome measure. With an α error probability of 0.05, the achieved power (1-β error probability) was 0.98.

## Discussion

This 3.0 T CMR study established reference mean values for myocardial T1ρ values across varying spin-lock frequencies and investigated gender effects. Key findings demonstrated that T1ρ values increased progressively with higher spin-lock frequency. Also, healthy females had significantly higher myocardial T1ρ values than healthy males. These data defined normal variation in quantitative T1ρ mapping and underscored the importance of standardizing spin-lock frequency and accounting for gender when applying T1ρ mapping clinically for myocardial tissue characterization.

This study presented the first investigation of T1ρ value variation at different spin-locking frequencies using a 3.0-T magnetic resonance imaging system. Previous work by Deng et al [20] characterized T1ρ value changes with slice position and gender on a 1.5-T system, providing reference mean values for clinical T1ρ sequence application. Building upon this, the high signal-to-noise ratio achievable with 3.0 T benefits T1ρ mapping investigations. However, the higher specific absorption rate (SAR) of 3.0 T versus 1.5 T necessitates caution regarding power consumption during continuous scanning. Each spin-locking frequency comes with its own set of technical challenges, including potential impacts on the signal-to-noise ratio and specific absorption rate considerations. Lower frequencies generally allow for lower SAR and safer imaging conditions, particularly important when scanning vulnerable populations or for longer durations. Different tissues and pathologies might respond differently to various spin-lock frequencies. For instance, fibrotic tissue, which has altered collagen content and water binding, may exhibit distinct relaxation behavior at different spin-lock frequencies [12, 21]. That means different pathological conditions might require different spin-lock frequencies to be optimally detected. As noted by Bustin et al [12], only a small range of spin-lock pulse amplitudes can be utilized clinically at 3.0 T. Furthermore, Wang et al [22] successfully utilized T1ρ imaging with a spin-lock frequency of 298 Hz to assess fibrosis in hypertrophic cardiomyopathy quantitatively. Therefore, appropriately reducing the spin-lock pulse amplitude can maintain long-term system stability without compromising diagnostic utility. Critically, a minimum amplitude of 300 Hz is recommended to suppress intrinsic T2 effects and optimize T1ρ map contrast, as advised by previous studies [21, 23].

Compared to published values at identical frequencies, our 3.0 T T1ρ values were slightly higher than Yin et al (300 Hz, T1ρ = 42.5 ± 3.4 msec) [24] and lower than Wang et al (400 Hz, T1ρ = 49.4 ± 2.6 msec) [25] and van Oorschot et al (500 Hz, T1ρ = 46.4 ± 1.8 msec) [19]. Interstudy differences may relate to variations in spin-lock duration, scanner type, and T1ρ preparation protocols. Standardizing T1ρ schemes could improve result comparability. T1ρ-weighted image contrast is dependent on both time of spin-lock (TSL) and frequency (FSL) of the spin-lock pulse [18]. The quantitative T1ρ value at a single spin-lock frequency can be derived from the formula: M(TSL) = M_0_${e}^{-\frac{{TSL}}{{T}_{1\rho }}}$ [26, 27], where M0 represents the initial longitudinal magnetization and TSL is the spin-lock pulse duration. This mathematical relationship indicates that the T1ρ value is solely determined by TSL when FSL is fixed. Some studies have suggested the potential to manipulate T1ρ image contrast by varying the spin-lock frequency [17, 21], at the same time, Koskinen et al [28] show that T1ρ decreases as the locking field B1 decreases, which just as T1 reduces with the static magnetic field B0. However, further investigations are required to fully characterize the dependence of T1ρ values on spin-lock parameters, which will facilitate the optimization of T1ρ-weighted pulse sequences for clinical applications.

Our findings demonstrated that T1ρ values were influenced by sex, consistent with previous observations by Deng et al [20]. Sex differences have also been noted in T1 and T2 mapping studies, suggesting potential biological factors underlying contrast variation [29]. However, the mechanisms through which gender impacts T1ρ values remain poorly elucidated. Considering known gender influences, incorporating sex as a variable in T1ρ sequence design and interpretation is advisable for future clinical applications in myocardial fibrosis imaging. Elucidating the biological and technical basis for gender contrast effects will inform optimized protocols tailored to the individual. Standardizing T1ρ methodologies accounting for sex differences could improve diagnostic consistency and accuracy.

In this study, high intra- and interobserver reproducibility, as well as excellent scan-rescan reproducibility, were observed for myocardial T1ρ measurements. The results indicated the robustness and reliability of our methodology and demonstrated the potential of this technique for stable and consistent quantification of myocardial characteristics in clinical and research settings.

### Limitations

We acknowledge the following limitations of our study. The disproportionate recruitment of female volunteers may introduce bias or imprecision in characterizing gender effects. The focused age distribution centered on young adults precluded assessments of age-related influences on T1ρ values. We could not explore potential variations with MRI scanners, T1ρ preparation pulse designs, or spin-locking durations as a single-center study. Follow-up inquiries with larger, more diverse cohorts are needed to fully delineate the impacts of participant demographics, acquisition protocols, and systems on quantifying T1ρ. Multicenter studies with balanced representation across sexes, ages, and protocols would enhance generalizability. Furthermore, correlating imaging findings with collateral measures of tissue composition could provide insight into the biological underpinnings of contrast.

## Conclusions

This study concluded that spin lock frequency and gender significantly influenced T1ρ relaxation time. T1ρ values increased with spin lock frequency, although the numerical differences were small. Furthermore, the T1ρ value was sex-dependent, with women exhibiting significantly higher values than men.

## Data Availability

Further information is available from the corresponding author on reasonable request.

## Abbreviations

CMR: Cardiac magnetic resonance
ICM: Ischemic cardiomyopathy
LGE: Late gadolinium enhancement
LV: Left ventricle
NICM: Non-ischemic cardiomyopathy
